# Pathways into homelessness: recently homeless adults problems and service use before and after becoming homeless in Amsterdam

**DOI:** 10.1186/1471-2458-9-3

**Published:** 2009-01-07

**Authors:** Igor R van Laere, Matty A de Wit, Niek S Klazinga

**Affiliations:** 1 GGD Municipal Public Health Service, Amsterdam, the Netherlands; 2 Academic Medical Centre, University of Amsterdam, Department of Social Medicine, Amsterdam, the Netherlands

## Abstract

**Background:**

To improve homelessness prevention practice, we met with recently homeless adults, to explore their pathways into homelessness, problems and service use, before and after becoming homeless.

**Methods:**

Recently homeless adults (last housing lost up to two years ago and legally staying in the Netherlands) were sampled in the streets, day centres and overnight shelters in Amsterdam. In April and May 2004, students conducted interviews and collected data on demographics, self reported pathways into homelessness, social and medical problems, and service use, before and after becoming homeless.

**Results:**

among 120 recently homeless adults, (male 88%, Dutch 50%, average age 38 years, mean duration of homelessness 23 weeks), the main reported pathways into homelessness were evictions 38%, relationship problems 35%, prison 6% and other reasons 22%. Compared to the relationship group, the eviction group was slightly older (average age 39.6 versus 35.5 years; p = 0.08), belonged more often to a migrant group (p = 0.025), and reported more living single (p < 0,001), more financial debts (p = 0.009), more alcohol problems (p = 0.048) and more contacts with debt control services (p = 0.009). The relationship group reported more domestic conflicts (p < 0.001) and tended to report more drug (cocaine) problems. Before homelessness, in the total group, contacts with any social service were 38% and with any medical service 27%. Despite these contacts they did not keep their house. During homelessness only contacts with social work and benefit agencies increased, contacts with medical services remained low.

**Conclusion:**

the recently homeless fit the overall profile of the homeless population in Amsterdam: single (Dutch) men, around 40 years, with a mix of financial debts, addiction, mental and/or physical health problems. Contacts with services were fragmented and did not prevent homelessness. For homelessness prevention, systematic and outreach social medical care before and during homelessness should be provided.

## Background

There is little evidence on good practice in caring for homeless people in the medical literature [1]. It has been reported that for homeless people life expectancy averages around 45 years, and that lack of access to health care services has too often proved a barrier to recovery, and, as a result, contributes to a downward spiral of deteriorating health and premature death [2]. Therefore, public services strategies should include homelessness prevention.

To prevent and reduce homelessness, strategies that address the general population and/or a targeted population could include housing benefits, welfare benefits, supplementary security income, supportive services for impaired or disabled individuals, programs to ameliorate domestic conflicts, programs to prevent evictions, discharge planning for people being released from institutions and (outreach) care programs for homeless populations [3,4]

Despite all these efforts and investments, and although there is broad consensus among policy makers and service providers that more resources and professional efforts should be dedicated to homelessness prevention, insufficient knowledge is available on *how *to accomplish this [3-5].

To identify starting points for homelessness prevention strategies in Amsterdam, the Netherlands, we have described in previous articles *evictions *from ones home as a major pathway of *how *people enter homelessness [6,7]. We demonstrated that evictions were a neglected public health problem. Despite knowledge about the underlying social and medical problems among households at risk, referrals to social and medical care are insufficiently used as a method to prevent eviction. Furthermore, we concluded that in Amsterdam nobody took the responsibility for the evicted households, predominantly due to rent arrears, whether they became homeless or not.

The absence of integrated social medical care results in a lack of assistance for recently evicted households, many of whom enter homelessness. Once homeless, people are responsible themselves in their search for specific services, organised alongside the mainstream service delivery system [8,9]. The lack of assistance for recently homeless people seems to be in concordance with a lack of knowledge of recently homeless people, related to their pathways into homelessness and their social and medical problems [3-5,9,10]

## Objective of this study

Regarding the lack of assistance for evicted households in Amsterdam, and contributing to the knowledge on recently homeless people and the development of prevention practice, for this study we tried to identify recently homeless adults, to explore 1) the pathways into homelessness, 2) the social and medical problems before and after becoming homeless, and 3) the contacts with social and medical services before and after becoming homeless.

## Methods

### Study population

Included in our study were recently homeless adults defined as persons, 18 years and older, who lost their house for the first time during the last two years (between April 2002 and April 2004) and who were legally staying in the Netherlands. The choice of the length of homelessness up to two years was intended to enhance the reliability of the information reported and to overcome problems of memory. To find locations to meet recently homeless adults, data on rough sleepers and visitors of day centres in Amsterdam were studied [11,12] Staff at one specific benefits provider for the homeless, at five day centres and at two emergency shelters were interviewed for information on their homeless visitors. After combining oral and written information, we decided to reach as many recently homeless adults as possible at locations recently homeless people tend to visit and where they could be approached for an interview. These were three gathering places for outreach soup distribution and popular street hangouts, one specific agency for benefits provision for the homeless, four emergency shelters and seven day centres with each over 450 visits a week. To keep a homogeneous sample, shelters for adolescents and families were not included. The study design did not need a process of ethical approval according to the Dutch Act on Medical Research.

In April and May 2004, interviews were conducted by ten undergraduate social science students. The students were familiar with approaching and interviewing homeless people. Interviewers underwent three training sessions on the process and quality of data registration, and all questionnaires were reviewed after the interviews. For every completed questionnaire students received twenty euros. Interviews lasted on average 45 minutes.

During a total of 40 occasions, at fourteen locations, between 4 and 38 homeless people were present at any moment (on average 25), of whom 125 homeless adults were eligible and participated in the study, by giving written consent for an interview and anonymous data analysis. Specific encouragement or incentives for homeless people to participate were not applied. None of the respondents were too intoxicated or too confused to be able to participate. During the interviews, on a separate list, the questionnaire number, a coded name and date of birth of participants were recorded to exclude doubling; two persons were interviewed twice and were excluded from analysis. Three questionnaires were excluded as the respondents were homeless for longer than two years. In total 120 questionnaires were included in the analysis.

### Collected data

Questionnaires for this study consisted of author-generated items. In consultation with city sociologists at the University of Amsterdam Department of Social Sciences, items of questionnaires used in follow up studies on rough sleepers were added [11]. Data were collected in a variety of areas addressing who, where, what, how and when questions following the process and antecedents of becoming homeless, self reported social and medical problems and contacts with social and medical services, before and after becoming homeless. Type of underlying problems chosen were based on the authors experience with providing outreach care to homeless people in Amsterdam over the last decade [13].

To find out *pathways into homelessness*, respondents were asked about their last housing condition and included composition of the household, type of housing, type of lessor, rent agreement and rent/income ratio. *Demographics *included sex, age and country of birth. For information on the *social and medical problems *before and after becoming homeless the following items were asked. Social problems were domestic conflicts (with household members, neighbours, landlords and/or services) and financial problems. For the latter data on financial debts, reasons for debts and type of creditors were collected. Medical problems included addiction to alcohol, drugs and gambling, mental health problems and physical health problems. Alcohol use could be scored as normal, excessive or extreme, according to the Garretsen scale [14]. Cocaine and/or heroine use could be more or less than 13 days a month; 13 days were chosen to exclude weekend users. Gambling could be absent or present. For mental problems no specific instruments or criteria were used for practical reasons. Respondents were asked if they felt depressed, fearful and/or confused. Physical problems and/or handicap were asked in an open question.

*Service contacts*, before and after becoming homeless, included social and medical services. Social services included social work, benefits agency, debt control agency, as well as shelters and day centres. Medical services included general practitioner, addiction service, mental health service and the GGD Municipal Public Health Service (safety net department and outpatient drug clinics) [13].

### Study assessments and analysis

Statistical analyses were performed using SPSS 14.0 and were mainly descriptive. The pathways into homelessness are described. Demographics, problems and service use are described and compared between the three main identified pathways into homelessness. Differences in the characteristics and underlying problems among homeless people following the different pathways are compared using chi-square and Fisher-exact tests for categorical variables and Wilcoxon median test for continuous variables. To identify independent factors associated with the specific pathways, logistic regression analyses was performed using backwards selection based on the loglikelihood ratio. In addition, logistic regression analyses was performed to study factors independently associated with the main problems identified in each pathway.

## Results

### Housing setting and pathways into homelessness

In table 1 the self reported housing setting and pathways into homelessness are shown. Before homelessness two thirds were living in a rented house. Thirteen respondents, out of the 120, mentioned never having lived independently; they had always been staying with family or friends. More than half had rented a house of a housing association (53%) and one third had rented privately (32%). The median rent price was 268 euros (range 0 – 1,000 euros), and the median gross salary was 809 euros (range 0 – 4,500 euros). Forty respondents had a rent/income ratio up to 30%, 33 up to 60%, 7 more than 60% and for 40 respondents this was not known.

**Table 1:** Self reported setting and pathways into homelessness#

*Type of housing (n = 115)*	*n*	*%*
own house	75	65
stay with family, friends or other	27	23
prison	6	5
abroad	3	3
hospital	2	2
hostel	2	2
*Type of tenant (n = 85)*		
housing association	45	53
private rent	27	32
subletting	10	12
other	3	4
**Pathways, how last housing lost? (n = 109)**		
eviction	41	38
relationship problems (left or sent away)	38	35
after prison	6	6
other reasons	24	22
*where last housing lost? (n = 117)*		
Amsterdam	95	81
rest of the Netherlands	16	14
abroad	6	5
*whereabouts after loss of housing? (n = 114)*		
family or friends	42	37
rough sleeping	30	26
shelter for the homeless	25	22
squads and garage boxes	8	7
abroad	5	4
clinic	4	4
*when homeless after loss of housing? (n = 98)*		
immediately	56	57
1 day – 1 week	8	8
1 week – 1 month	8	8
1–3 months	13	13
3–6 months	8	8
> 6 months	5	5
*how long currently homeless? (n = 120)*		
< 1 month	11	9
1–3 months	11	9
3–6 months	42	35
6–12 months	30	25
12–18 months	16	13
18–24 months	10	8

# 120 recently homeless people in Amsterdam who lost their house for the first time between April 2002–April 2004.

When asked *how *respondents lost their last housing, answered by 109 respondents, the three main pathways were *evictions *(38%), leaving ones house or being send away by others due to *relationship problems *(35%) and *other reasons *(28%). Among 38 respondents who were homeless due to relationship problems, (of whom one third had a rent contract in their own name), 4 had left on their own initiative and 34 were sent away by household members (partner 22, parents 6 and roommates 6). Among other reasons, 6 mentioned they had lost their house while doing time in prison. Four out of five had become homeless in Amsterdam. After loss of last housing, 57% reported immediate homelessness, and 86% reported being on the streets within three months. The median length of homelessness was six months (23 weeks).

### Demographics and household composition

In table 2 demographics and household composition related to pathways through which people became homeless are shown. Compared to the relationship group, the eviction group was slightly older (average age 39.6 versus 35.5 years; p = 0,08), living single more often (p = 0,000), and belonged to one of the major migrant groups more often (p = 0.025). The total average age for both sexes was 38 years, the range for males was 18–67 years, and for females 19–50 years.

**Table 2:** Demographics and household composition related to pathways into homelessness#

	total (n = 120)		eviction (n = 41)		relationship (n = 38)		other (n = 30)	
Demographics	n	%	n	%	n	%	n	%
*sex*								
male	105	88	38	93	33	87	26	87
female	15	12	3	7	5	13	4	4

*Age in years*								
18–29	39	33	8	20	13	35	10	33
30–39	30	25	15	38	11	30	8	27
40–49	26	22	8	20	7	19	6	20
50–59	14	12	6	15	4	11	3	10
60–67	9	8	3	8	2	5	3	10

*Country of birth*								
Netherlands	58	48	15	38	18	47	21	70
Surinam/Antilles/Morocco	24	20	13	33*	6	16	4	13
Other	37	31	12	30	14	37	5	17

*composition of household*								
single	46	44	26	63**	3	10	12	48
with parents	6	6	1	2	3	10	2	8
with partner	22	21	4	10	13	43	4	16
with partner and children	18	17	4	10	8	27	4	16
single parent	3	3	2	5	0	0	1	4
with other adult	10	9	4	10	3	10	2	8

# 120 recently homeless people in Amsterdam, who lost their house for the first time between April 2002–April 2004.

* p = 0.025; ** p = 0.000

### Pathways and problems

Self reported problems before homelessness related to pathways into homelessness are shown in table 3. Social problems were present in 81% of the total group, and medical problems in 76%. In the total group, before homelessness, almost two thirds (62%) had both social and medical problems (not in table 3). As expected, regarding pathways and social problems, the eviction group had significantly more often financial debts than the relationship group (p = 0,009). Logistic regression analyses showed that the only factor independently associated with financial debts were alcohol problems (OR 7.0 (95% CI: 2.0–25.0). Also in the relationship group almost half reported financial debts. The relationship group reported more domestic conflicts than the other groups (p < 0.001). Domestic conflicts were more common among those between 18–29 years and those 60 years and older, and among respondents not born in the Netherlands. Underlying social or medical problems were not significantly associated with domestic conflicts.

**Table 3:** Self reported problems before homelessness related to pathways into homelessness#

	total (n = 120)		eviction (n = 41)		relationship (n = 38)		other (n = 29)	
Reported problems before homelessness	n	%	n	%	n	%	n	%
Social problems (n = 109)	88	81	35	85	32	84	21	70
financial debts	73	61	33*	81	18	47	18	62
domestic conflicts	55	46	18	44	29**	76	6	21
Medical Problems (n = 109)	83	76	32	78	30	79	21	70
Addiction total	57	48	24	59	17	45	10	35
alcohol ##	26	22	12***	29	5	13	4	13
drugs	37	31	12	29	15	40	6	21
cocaine	33	28	10	24	14	37	6	21
heroin	14	12	5	12	6	16	1	3
gambling	22	18	10	24	5	13	4	14
Mental problems	67	56	26	63	20	53	16	53
depressed	61	51	24	59	18	47	14	47
fearful	29	24	15	37	6	16	5	17
confused	23	19	8	20	6	16	7	23
Physical problems	26	22	7	17	10	26	8	28

# 120 recently homeless people in Amsterdam who lost their house for the first time between April 2002–April 2004.

## according Garretsen scale [14]

*p = 0.009; **p < 0.001; ***p = 0.048

Regarding pathways and medical problems, the eviction group reported more extreme alcohol problems than the relationship group (p = 0.048). Drug problems, mainly cocaine use, tended to be more common in the relationship group compared to the eviction group, although not significantly. In all groups more than half reported mental health problems.

Not reported in table 3, among 73 respondents with debts, the main reasons for debts were loss of job and/or chronic shortage of income (49%), buying drugs (18%), gambling (10%), and other reasons such as fines, order by credit and health costs (23%). Of 22 gamblers, 16 respondents reported financial debts. Of 73 respondents with debts, 16 reported gambling. The majority of creditors were banks (35%), landlords (34%), energy companies (18%) and family members (9%). The median debt was 5,000 euros (range 400 – 400,000 euro). One person had left a mortgaged house leaving a 400,000 euros debt.

In table 4 problems before and after becoming homeless are shown. Financial problems before homelessness (61%) were not solved during homelessness, when even more respondents reported debts (68%). The overall addiction rate had decreased from 48% before to 20% after becoming homeless, due to less excessive and extreme use of alcohol, less use of heroin, and less use of cocaine. The self reported gambling rate decreased from 18% before to 3% after becoming homeless. During homelessness only a few individuals began substance use for the first time. Feelings of being depressed, fearful and confused were frequently reported before (56%) and after (63%) becoming homeless, and in both periods almost one quarter reported physical problems or a handicap.

**Table 4:** Self reported problems before and after becoming homeless#

	*before*		*after*	
Reported problems	n	%	n	%
*Social problems*	88	81	82	68
financial debts	73	61	82	68
domestic conflicts	55	46	-	-
*Medical Problems*	83	76	89	74
Addiction total	57	48	22	20
alcohol*	26	22	9	8
excessive	15	13	3	3
extreme	11	9	6	5
drugs	37	31	17	14
cocaine	33	28	16	13
< 13 days per month	23	19	8	7
13+ days per month	10	8	8	7
heroin	14	12	6	5
< 13 days per month	6	5	2	2
13+ days per month	8	7	4	3
gambling	22	18	4	3
Mental problems	67	56	75	63
depressed	61	51	68	57
fearful	29	24	24	20
confused	23	19	22	18
Physical problems	26	22	29	24
*Total social and medical*	76	63	63	55

# 120 recently homeless people in Amsterdam who lost their house for the first time between April 2002–April 2004.

* according Garretsen scale [14]

### Pathways and service use

The self reported service use for social and medical problems before and after becoming homeless is shown in table 5. Despite the fact that a combination of debts, addiction and/or mental health problems were often reported, contacts with social services were low and with medical services even lower. Among the contacts with medical services the general practitioner played a minor role.

**Table 5:** Self reported service use before and after becoming homeless#

	*before*		*after*	
Service use	n	%	n	%
*social services total*	45	38	100	83
social work	26	22	44	37
benefit agency	18	15	49	41
debt control agency	17	14	12	10
shelters and daycentres	22	18	73	61

*medical services total*	32	27	32	27
general practitioner	6	5	13	11
addiction service	12	10	9	8
mental health service	13	11	7	6
GGD public health service*	13	11	14	12

*all services*	65	57	106	93

#120 recently homeless people in Amsterdam who lost their house for the first time between April 2002–April 2004.

*GGD safety net department and outpatient drug clinics [13].

Regarding pathways and social problems, the eviction group reported more contacts with debt control services than the relationship group (33/41 = 81% versus 18/38 = 47%; p = 0.009, not in table). Despite these contacts they did not keep their house. Regarding pathways and medical problems no significant differences in service use between pathway groups were found. Before homelessness, of 86 respondents who reported a medical problem, 47 did look for some sort of medical service and 39 did not feel the need. Reasons mentioned for not perceiving the need for medical support were e.g. "I don't need help", "I solve my own problems", "I don't have an addiction problem", "I don't see how they can help me", "I don't know where to go", "they ask too many questions" and "services are slow".

### How recently homeless people envision better services and their biggest dream

We asked recently homeless people about their ideas how to improve assistance. In general, the majority of respondents mentioned that they wished that the city provides a one stop comprehensive service for social and medical problems, active assistance for red tape and financial management, and fast tracking towards (guided) housing and jobs. Respondents said e.g.: "you need to be verbally strong to succeed at services", "social and financial support should be much faster", "I wish clear information where to go for what problem", "services should work together". Other answers were: "If I had help before I became homeless....", "I try to be nice, but they are rude", and "they should offer help for normal homeless people".

What is your biggest dream? Almost all wanted a house, a normal life with family contacts and/or a job. Respondents said e.g. "I hope they give me benefits in the future", "to see my daughter", "a safe place", "a house within a few months, and celebrate Christmas with friends at home". Other answers were: "that they do more for homeless people who do not take drugs", "I do not have dreams, I gave up hope a long time ago" and one man was dreaming of "a shower and clean clothes".

## Discussion

For the homelessness prevention practice, we aimed to discover the *sources of homelessness; *defined as the factual pathway that leads to an (official) forced or voluntary displacement from ones home or facility. Therefore, we explored the pathways people took into homelessness and compared the characteristics, problems and service use per pathway taken. In our approach, we focus on the detection of underlying problems, that services should respond to, rather than exploring the reasons why the underlying problems exist. Knowledge of the characteristics and problems of people who follow different pathways into homelessness should contribute to timely detection of vulnerable people who might step into homelessness.

We identified 120 recently homeless people in Amsterdam to explore their pathways into homelessness, problems and service use, before and after becoming homeless. The main pathways into homelessness reported were *evictions from ones home *(38%), *relationship problems *that lead to leaving a home or being sent away by household members (35%), *leaving prison *(6%) and various other reasons (22%). These pathways into homelessness are consistent with those known in the literature [4-10,15,16]. However, the figures in this sample can not be compared with those found by others due to varying settings, definitions and methodology. For comparison, the factual pathways into homelessness, the key causes, underlying contextual factors and triggers need to be disentangled [4,5,9].

Not surprisingly, the characteristics of the recently homeless people in our study show more similarities than differences with those found among the majority of households at risk of eviction (due to rent arrears and nuisance), rough sleepers, shelter users and homeless adults visiting outreach medical care facilities in Amsterdam [6,11,17-19]. The profile of the majority of the homeless in Amsterdam is comparable with those in cities abroad [10,20-22].

In all pathway groups almost two thirds reported a combination of social and medical problems. Those who were homeless after eviction did belong to a major migrant group more often, were slightly older, were more often living single, had more financial problems and more alcohol problems, than the other groups. Those who were homeless due to relationship problems were slightly younger, had more domestic conflicts and tended to report more drug (cocaine use) problems, than the others groups.

Gambling, as a known source of debts and financial difficulties, was reported by 24% among those evicted and 13% among those who had lived with others. In Melbourne, Australia, before homelessness, among 93 older homeless men, gambling was reported by 46% among those who were living alone and 28% among those living with others [4,16]. In Amsterdam, gambling was hardly mentioned by employees of housing associations handling rent arrears and by employees in nuisance control care networks handling nuisance, when asked to report problems among households at risk of eviction [6]. Service providers should be alert for gambling problems among mostly single men at the brink of homelessness due to financial difficulties.

Furthermore, regarding medical support before homelessness, for all pathways, the general practitioner, as a gatekeeper for addiction, mental and physical health problems, played a marginal role in providing care, which was also found among households at risk of eviction in Amsterdam [7]. For those at risk of homelessness with silent and/or non-self perceived health needs, 39 out of 86 who reported a medical problem, a sharp decrease of home visits carried out by general practitioners might be unfavourable [8,23]. Specifically, if no alternative social medical care at home is provided, and lessons how to integrate care for those in highest need have to be learned in the streets [25]. Therefore, rent arrears and nuisance can serve as signals to explore underlying problems by outreach support [6,7].

After becoming homeless, most problems identified before homelessness were also reported to exist afterwards, except for substance use and gambling, which had decreased significantly. The fact that many recently homeless had sought social care and were willing and capable of placing their addiction more in the background, is an indication of the motivation within this group to turn their situation around. The addiction decrease could be an indication that in the first homeless period the scarce financial means are being used mainly for subsistence. This moment should be an entry point for service providers to actively guide the recently homeless towards rehabilitation. Although validated diagnostic mental health tools were not used, by often reporting mental health problems many respondents did not seem satisfied with their mental health condition and/or situation. For recently homeless people staying in the same shelters and day centres together with the long-term homeless might have a numbing effect on a positive attitude towards rehabilitation [26,27].

The strength of this study is that we had good access to key informants and the locations where recently homeless people tend to gather. We obtained a high response rate among the recently homeless people who were approached for an interview. This study involved two principal limitations. First, our data regarding medical problems were based on self-reported information. Specifically for psychiatric problems diagnostic or clinical instruments were not used, therefore data can not be compared with other studies. Furthermore, some respondents mentioned having trouble remembering the number of services they had used over time. Second, a random sample of the recently homeless could not be drawn since the duration of homelessness is not registered at day centres and shelters, and not for those not using these facilities. Following our experience with homeless care, we believe that the data are valid and can be generalised for the total recently homeless population in Amsterdam.

### Homelessness prevention strategies

Scholars in Australia, England and the US have described multiple obstacles for homelessness prevention strategies and the evaluation of prevention programs [3-5]. Regarding causes of homelessness, most cases involved personal problems and incapacities, policy gaps and service delivery defects. Crane et al. found that vulnerable people were being excluded because health and welfare services did not have the responsibility or resources to search for people with unmet treatment or support needs [4,5]. Furthermore, evaluation of homelessness prevention programs are hampered e.g. by fragmented and provision driven data registration [3].

In Amsterdam, several strategies to prevent and reduce homelessness have been implemented, since our study was executed in 2004. The Amsterdam Welfare and Care department promotes an integrated approach by housing, social and medical services to take responsibility in actively assisting vulnerable citizens with unmet support needs. This strategy is in concordance with the wishes and dreams of the majority of the recently homeless in our study. Since 2007 service providers are being trained for this approach to learn how to explore problems and pathways towards shared assistance. Furthermore, with substantial national and local financial support, services are able to expand their activities. More guided living options in the social housing sector (75% of the total housing stock in Amsterdam) are being offered, more integrated one stop social medical service units will be build, and the number of beds in shelters, addiction and mental health care facilities are being increased [28].

Regarding the three pathways into homelessness of the recently homeless people in our study, we reflect and comment on the existing strategies in Amsterdam.

1) *Eviction from ones home *was the main source of homelessness. Per year more than 1,400 households are being evicted in Amsterdam [6]. To decrease the number of evictions, the existing outreach networks respond to persistent rent arrears and nuisance, as signals to be picked up by housing associations and landlords, to be shared with social services. In response, during a house visit underlying problems, such as gambling and medical problems, and unmet support needs are being explored [6,7,28]. Based on our previous studies on evictions and current findings, we suggest that assistance should explicitly be applied to low income single men, with underlying financial problems, addiction and/or mental and/or physical health problems. As among these high risk men a mix of social and medical problems is to be expected, social and medical workers should be trained to systematically approach and guide the underlying problems to keep these men at home [6,7,25].

2) *Relationship problems *that lead to leaving a house was the second source of homelessness. Prevention strategies might be difficult to design. However, underlying problems and service use are also prevalent among this high-risk group. Alertness of social and medical services could be the way to identify this high risk group for preventive actions. Services should know their clients and should (be trained to) be sensitive for signals of vulnerability. These signals should be detected with a few additional questions related to how a person is coping with daily living, household management, income and debts (alcohol, cocaine and gambling), and should actively be shared among disciplines [4,5,21]. In health care settings medical professionals, and the general practitioner in particular, do have the opportunity and responsibility to diagnose social disease (such as poverty and imminent homelessness), that intrinsically interacts with medical disease, and actively ask for social assistance in response [5,29].

3) *Leaving prison *was the third source of homelessness, among various other reasons. In the Netherlands, when people stay in prison for a certain period of time welfare benefits are being terminated. Data on the number of people that did pay rent off welfare benefits before they went to prison are not being collected. Nor data on the number of people that lost their house during time in prison because nobody assisted in paying the rent at home, and, as a consequence, became homeless after leaving prison. However, in Amsterdam, vulnerable inmates and multiple offenders are actively being followed up and assisted to anticipate housing, income and care after prison [28].

Furthermore, *to prevent long term homelessness*, new arrivals in the homeless circuit, at places the homeless tend to gather, are actively being identified and fast tracked along social and medical services, as the motivation to turn their situation around is expected to be a crucial entry point towards rehabilitation. For this strategy, social and shelter services aim to converge their intake procedures in a central shelter unit, where (recently) homeless people can undergo a social medical assessment and be guided towards problem oriented housing and care. Among the services for the poor and underserved, the GGD Municipal Public Health Service is operating as the central field director to monitor strategies to further prevent and reduce homelessness in Amsterdam [28]. New evaluations should demonstrate whether the present situation has improved compared to our findings in 2004.

## Conclusion

Among recently homeless adults in Amsterdam, the main pathways into homelessness reported were evictions, relationship problems and leaving prison. In all pathways, the recently homeless fit the profile of the majority of the total homeless population in Amsterdam: single men, around 40 years, with a mix of debts, domestic conflicts, addiction, mental and/or physical health problems. Regarding service use before becoming homeless, and regardless the pathway taken, more than half reported contacts with social and/or medical services that did not prevent homelessness. During homelessness only contacts with social work and benefit agencies increased, contacts with medical services remained low. For homelessness prevention, systematic and integrated social medical care before and during homelessness should be provided.

## Competing interests

The authors declare they have no competing interests. No external funding was provided for this research.

## Authors' contributions

All authors contributed to the conceptualisation of the paper. IvL contributed to the study design and implementation, and wrote the manuscript. MdW contributed to the study design and implementation, analysed the data and assisted in writing the manuscript. NK contributed to the manuscript design and assisted in writing the manuscript.

## Pre-publication history

The pre-publication history for this paper can be accessed here:

http://www.biomedcentral.com/1471-2458/9/3/prepub

## References

[B1] Hwang SW Tolomiczenko G Kouyoumdjina FG Garner RE Interventions to Improve Health of the Homeless: a systematic review Am J Prev Med 2005 29 4 311 19 10.1016/j.amepre.2005.06.017 16242595

[B2] O'Connell JJ Premature Mortality in Homeless Populations: A Review of the Literature 2005 Nashville, USA: National Health Care for the Homeless Council, Inc http://www.nhchc.org/PrematureMortalityFinal.pdf

[B3] Shinn M Baumohl J Hopper K The prevention of Homelessness Revisited Analyses of Social Issues and Public Policy 2001 95 127 10.1111/1530-2415.00006

[B4] Crane M Byrne K Fu R Lipmann B Mirabelli F Rota-Bartelink A Ryan M Shea R Watt H Warnes AM The causes of homelessness in later life: findings from a 3-nation study J Gerontol B Psychol Sci Soc Sci 2005 60 3 S152 9 15860792 10.1093/geronb/60.3.s152

[B5] Crane M Warnes AM Fu R Developing homelessness prevention practice: combining research evidence and professional knowledge Health Soc Care Community 2006 14 2 156 66 10.1111/j.1365-2524.2006.00607.x 16460365

[B6] van Laere IRAL de Wit MAS Klazinga NS Evictions as a neglected public health problem: characteristics and risk factors of households at risk in Amsterdam in press 10.1177/1403494809343479 19666669

[B7] van Laere IRAL de Wit MAS Klazinga NS Evaluation of the signalling and referral system for households at risk of eviction in Amsterdam Health Soc Care Community 2008 in press 10.1111/j.1365-2524.2007.00790.x 19125966

[B8] Plumb JD Homelessness: care, prevention, and public policy Ann Intern Med 1997 126 12 973 5 Review 9182475 10.7326/0003-4819-126-12-199706150-00007

[B9] Anderson I Baptista J Wolf J Edgar B Benjaminsen L Sapounakis A Schoibl H The changing role of service provision: barriers of access to health services for homeless people 2006 Brussels: Feantsa, European Observatory on Homelessness 8 10 http://www.feantsa.org/files/transnational_reports/2006reports/06W3en.pdf

[B10] Schanzer B Dominguez B Shrout PE Caton CL Homelessness, health status, and health care use Am J Public Health 2007 97 3 464 9 10.2105/AJPH.2005.076190 17267724 PMC1805022

[B11] Deben L Rensen P Duivenman R [The homeless at night in Amsterdam 2003] 2003 Amsterdam: Aksant [Dutch]

[B12] [Exploration of the provision of drop in day centres for the homeless in Amsterdam] Amsterdam Gemeentelijke Dienst Maatschappelijke Ontwikkeling (DMO) afdeling Maatschappelijke en Gezondheidszorg 2003 [Dutch]

[B13] van Laere IRAL Outreach Medical Care for the Homeless in Amsterdam. Ambulatory Medical Team: the years 1997–2004 2005 Amsterdam: GGD Municipal Health Service

[B14] Garretsen HFL [Problem drinking: prevalence, associated factors and prevention: theoretical considerations and research in Rotterdam] Thesis 1983 Lisse: Swets & Zeitlinger [Dutch]

[B15] Crane M Warnes AM Evictions and Prolonged Homelessness Housing Studies 2000 15 5 757 773 10.1080/02673030050134592

[B16] Rota-Bartelink A Lipmann B Causes of homelessness among older people in Melbourne, Australia Australian and New Zealand Journal of Public Health 2007 31 3 252 8 10.1111/j.1467-842X.2007.00057.x 17679244

[B17] Buster MCA van Laere IRAL [Dynamics and problems among homeless people using shelters in Amsterdam] 2001 Amsterdam: GG&GD [Dutch]

[B18] van Laere IRAL Buster MCA [Health problems of homeless people attending the outreach primary care surgeries in Amsterdam] Ned Tijdschr Geneeskd 2001 145 1156 60 [Dutch] 11433664

[B19] Sleegers J Similarities and differences in homelessness in Amsterdam and New York City Psychiatr Serv 2000 51 1 100 4 10647141 10.1176/ps.51.1.100

[B20] Morrell-Bellai T Goering PN Boydell KM Becoming and remaining homeless: a qualitative investigation Issues Ment Health Nurs 2000 21 6 581 604 10.1080/01612840050110290 11271135

[B21] Goering P Tolomiczenko G Sheldon T Boydell K Wasylenki D Characteristics of persons who are homeless for the first time Psychiatr Serv 2002 53 11 1472 4 10.1176/appi.ps.53.11.1472 12407279

[B22] Fountain J Howes S Marsden J Strang J Who uses services for homeless people? An investigation amongst people sleeping rough in London Journal of Community & Applied Social Psychology 2002 12 1 71 75 10.1002/casp.654

[B23] Berg MJ van den Cardol M Bongers FJ de Bakker DH Changing patterns of home visiting in general practice: an analysis of electronic medical records BMC Fam Pract 2006 7 58 10.1186/1471-2296-7-58 17044914 PMC1624836

[B24] Allen T Improving housing, improving health: the need for collaborative working Br J Community Nurs 2006 11 4 157 161 16723906 10.12968/bjcn.2006.11.4.20836

[B25] van Laere IRAL Withers J Integrated care for homeless people – sharing knowledge and experience in practice, education and research: Results of the networking efforts to find Homeless Health Workers Eur J Public Health 2008 18 1 5 6 10.1093/eurpub/ckm107 18211914

[B26] O'Toole TP Gibbon JL Hanusa BH Fine MJ Preferences for sites of care among urban homeless and housed poor adults J Gen Intern Med 1999 14 10 599 605 10.1046/j.1525-1497.1999.09258.x 10571704 PMC1496749

[B27] Daiski I Perspectives of homeless people on their health and health needs priorities J Adv Nurs 2007 58 3 273 81 10.1111/j.1365-2648.2007.04234.x 17474916

[B28] [Off the streets: better care, less homelessness and less nuisance. Changes in service delivery for the years 2007–2010] 2007 Amsterdam: Gemeente Amsterdam, Dienst Zorg en Samenleven [Dutch]

[B29] van Laere IRAL Caring for homeless people: can doctors make a difference? Br J Gen Pract 2008 58 550 367 10.3399/bjgp08X280335 18482499 PMC2435679

